# Liposomal Nano-Curcumin Attenuates Cisplatin-Induced Hepatotoxicity in Rats with Concomitant Regulation of Wnt/β-Catenin/GSK-3β and Nrf2 Signaling

**DOI:** 10.3390/biomedicines14091907

**Published:** 2026-08-26

**Authors:** Qamraa H. Alqahtani, Muhammad Atteya, Tahani A. Al-Matrafi, Hamad M. Alqahtani, Esraa Kamal, Iman H. Hasan

**Affiliations:** 1 Department of Pharmacology and Toxicology, College of Pharmacy, King Saud University, P.O. Box 22452, Riyadh 11495, Saudi Arabia; 2 Department of Anatomy, College of Medicine, King Saud University, P.O. Box 2925, Riyadh 11461, Saudi Arabia; 3 Department of Pharmaceutics, College of Pharmacy, King Saud University, P.O. Box 22452, Riyadh 11495, Saudi Arabia

**Keywords:** nano-curcumin, cisplatin, hepatic toxicity, Wnt/β-catenin, GSK-3, Nrf2

## Abstract

**Objective**: This research examined the effectiveness of liposomal N-Curcumin (N-Cur) against Cis-diamminedichloroplatinum (CDDP)-induced hepatotoxicity, specifically examining its ability to influence the Wnt/β-catenin/GSK-3 pathway and associated molecular markers. **Methods**: Male Wistar rats were treated for 14 days with oral N-Cur (80 mg/kg) and with a single intraperitoneal dose of CDDP (7 mg/kg) administered on day 7. Hepatic integrity was assessed through liver injury markers, histopathological examination, and biochemical analysis of oxidative stress (SOD, GSH, and MDA), inflammation (IL-10, TNF-α, CRP, and NF-κB p65), and signaling protein expression (β-catenin, Nrf2, and GSK-3). **Results**: CDDP administration resulted in significant hepatic damage, characterized by elevated injury markers and distorted tissue architecture. It induced severe oxidative stress (increased MDA; decreased GSH and SOD) and a robust inflammatory response. At the molecular level, CDDP suppressed the cytoprotective β-catenin and Nrf2 pathways while increasing GSK-3. Conversely, N-Cur treatment effectively reversed these pathological shifts by restoring antioxidant defenses, inhibiting pro-inflammatory mediators, and normalizing the Wnt/β-catenin/GSK-3 signaling axis. **Conclusions**: Liposomal N-Cur demonstrates significant potential as a hepatoprotective agent when administered concomitantly with CDDP chemotherapy. N-Cur mitigates oxidative damage and inflammation, thereby preserving hepatic function during CDDP-based chemotherapy. In addition, these favorable effects were associated with modulation of the Wnt/β-catenin/GSK-3 and Nrf2 pathways.

## 1. Introduction

Cisplatin, also known as cis-diamminedichloroplatinum (II) (CDDP), is a commonly utilized chemotherapeutic drug, especially in solid tumors, including lung, testicular, bladder, cervical, ovarian, and head and neck cancers. Its high potency in improving survival outcomes and cure rates has led to its incorporation into standard-of-care treatment protocols in many malignancies [1]. Nonetheless, the effectiveness of CDDP in clinical settings is often constrained by its organ toxicities that depend on its dose, including nephrotoxicity, hepatotoxicity, ototoxicity, and cardiotoxicity, which may necessitate dose reduction or treatment cessation [2].

The hepatotoxic effects of CDDP are mediated through multiple interconnected mechanisms, including direct DNA damage via the formation of DNA adducts, ultimately triggering hepatocyte apoptosis [3]. Moreover, oxidative stress plays a key role in liver damage caused by CDDP, since CDDP increases the generation of reactive oxygen species (ROS), which surpass antioxidant defenses and lead to lipid peroxidation (LPO), as well as oxidative harm to proteins and DNA [4]. Redox imbalance is frequently accompanied by a marked inflammatory response, manifested as an elevation of C-reactive protein (CRP) and numerous pro-inflammatory mediators, including interleukin-10 (IL-10) and tumor necrosis factor-alpha (TNF-α), which ultimately contribute to hepatocellular damage and the elevation of serum transaminases [5]. Alongside this, nuclear factor kappa-B (NF-κB) activation amplifies oxidative stress, creating a cycle of inflammation and cellular damage. This interplay leads to hepatocyte apoptosis, fibrosis, and liver dysfunction [6].

Moreover, apoptosis plays a critical role in CDDP-induced hepatotoxicity, as accumulating evidence indicates that cisplatin activates the intrinsic apoptotic pathway in hepatic tissue, characterized by upregulation of pro-apoptotic proteins (e.g., Bax), downregulation of anti-apoptotic Bcl2, augmentation of caspase 3 activity, and consequent disruption of normal liver architecture [7]. Recent studies also highlight the involvement of endoplasmic reticulum stress in amplifying oxidative stress-induced hepatocyte apoptosis and worsening of CDDP-induced liver damage [8].

Emerging evidence indicates that Wnt/β-catenin signaling is crucial in CDDP-induced hepatotoxicity. Normally, this pathway supports liver health and cell survival, but CDDP exposure inhibits β-catenin activity, leading to reduced hepatocyte survival and regeneration [9]. This inhibition is linked to increased activity of glycogen synthase kinase-3 (GSK-3), which promotes β-catenin degradation. Under oxidative stress, heightened GSK-3 activity favors apoptosis and worsens liver injury. Overall, dysregulation of the Wnt/β-catenin/GSK-3 axis leads to impaired tissue repair and increased liver dysfunction, suggesting it as a potential therapeutic target for hepatoprotection [10]. Furthermore, the association between oxidative stress, inflammation, and Wnt/β-catenin signaling exacerbates hepatic damage, as ROS and inflammatory mediators suppress β-catenin while promoting GSK-3 activation. Collectively, dysregulation of the Wnt/β-catenin/GSK-3 axis contributes to impaired tissue repair, increased apoptosis, and progression of CDDP-induced liver dysfunction, highlighting this pathway as a promising therapeutic target for hepatoprotection.

A growing body of evidence suggests that both pharmacological and natural agents can reduce CDDP-induced hepatotoxicity by decreasing oxidative stress, inflammation, and apoptosis while maintaining antitumor activity [11]. Nevertheless, there remains a compelling need to identify effective, safe, well-characterized hepatoprotective agents that can be added to CDDP-containing regimens to improve treatment tolerance. In this context, curcumin, a polyphenolic constituent of *Curcuma longa*, emerges as a promising agent that exhibits anti-inflammatory, antioxidant, and anti-apoptotic properties and has been extensively studied in various pathological conditions such as neurodegeneration and hepatic disorders. Preclinical studies showed that curcumin attenuates CDDP-induced hepatic injury by alleviating oxidative stress, controlling inflammatory cytokine production and inhibiting apoptotic proteins, resulting in normalization of the liver’s function and architecture [12]. Moreover, recent experimental studies have confirmed the hepatoprotective effects of curcumin against cisplatin-induced liver injury through modulation of oxidative stress, inflammation, and apoptosis [13]. Beyond its hepatoprotective effects, curcumin also exhibits anticancer activities, including those against hepatocellular carcinoma, through modulation of cell proliferation, inflammatory mediators, and autophagy, which further underscores its importance as a multipurpose agent in oncology [14].

Despite its pharmacological potential, the clinical utility of native curcumin is limited by various unfavorable properties, including poor aqueous solubility and low oral bioavailability, as well as rapid metabolism and restricted tissue distribution [15]. To overcome these limitations, several formulation strategies have been developed, including nano-curcumin and liposomal curcumin, to improve solubility, systemic exposure, tissue distribution, and pharmacological efficacy. Nano-curcumin refers to curcumin engineered at the nanoscale to enhance dispersibility, cellular uptake, and bioavailability, whereas liposomal curcumin consists of curcumin encapsulated within phospholipid vesicles that protect it from degradation and facilitate cellular delivery. Overall, nanoformulations improve aqueous dispersibility, cellular uptake, tissue distribution, and bioavailability. Liposomal systems further enhance solubility and stability, potentially prolong systemic exposure, and promote intracellular delivery via membrane fusion and/or endocytosis [16]. Accordingly, liposomal nano-curcumin integrates the advantages of nanoscale formulation with the protective and delivery properties of liposomal encapsulation.

Within this context, nano-curcumin preparations have shown improved protection against chemotherapy-induced toxicities [17]. Recent studies have further supported these findings, emphasizing the enhanced bioavailability and therapeutic potential of nanoformulated curcumin, as well as its capacity to reduce oxidative stress, inflammation, and drug-induced hepatic injury compared to traditional curcumin formulations [16].

A previous study demonstrated that nano-curcumin reduced cisplatin (CDDP)-induced hepatotoxicity by suppressing oxidative stress, inflammatory responses, and hepatic fibrosis, supporting its potential as a hepatoprotective agent [18]. However, although conventional curcumin and nano-curcumin have been evaluated in experimental models of CDDP-induced liver injury, data on liposomal nano-curcumin remain limited. In particular, the molecular mechanisms underlying its potential hepatoprotective effects in CDDP-induced hepatic injury have not been fully defined. Curcumin has been linked to antioxidant pathways, including Nrf2 activation, as well as modulation of signaling networks involved in oxidative stress and cell survival [19]. However, whether liposomal nano-curcumin can concurrently modulate the Wnt/β-catenin/GSK-3β and Nrf2 pathways in CDDP-induced hepatotoxicity is still unclear, representing a key knowledge gap addressed in this study.

Therefore, this study aimed to evaluate the hepatoprotective effects of liposomal nano-curcumin against CDDP-induced liver toxicity and to investigate its impact on the Wnt/β-catenin/GSK-3β and Nrf2 signaling pathways. We also assessed its ability to mitigate oxidative stress, inflammation, and hepatocellular damage associated with CDDP exposure. By examining these pathways in parallel, this work provides further insight into the molecular basis of liposomal nano-curcumin-mediated hepatoprotection and extends current understanding of its role in CDDP-induced liver injury.

## 2. Materials and Methods

### 2.1. Chemicals and Reagents

CDDP (50 mg/100 mL) was sourced from EBEWE Pharma (Unterach am Attersee; Austria) (GmbH. Nfg. KG). Liposomal N-Cur was acquired from Lipolife (Essex, UK), while carboxymethylcellulose (CMC) and liver function tests (aspartate aminotransferase (AST) and alanine aminotransferase (ALT)) were obtained from an Elab-science ELISA kit (Wuhan, China; Cat. no. E-EL-R1253). Lactate dehydrogenase (LDH) was provided by Randox kits (Crumlin, Northern Ireland, UK; Cat. no. LD3818). Tumor necrosis factor (TNF-α) and interleukin-10 (IL-10) were obtained from MyBioSource ELISA kits (San Diego, CA, USA; Cat. no. MBS733016, MBS2020828, respectively), along with CRP (RAB0097, Sigma Aldrich, St. Louis, MO, USA) and the Nrf2 ELISA (Shanghai, China; BT LAB, Cat. NO. E1083Ra). GSH, MDA, and SOD levels were measured with Biodiagnostic kits (Giza, Egypt; Cat. no. TA2511, MD2528, SD2521, and CA2517, respectively). Antibodies for β-catenin (Cat. NO. MA1-301), GSK-3 (Cat. NO. 44-610), and Nrf2 (Cat. NO. PA5-67520) were acquired from Thermo Fisher Scientific Inc. (Waltham, MA, USA). Additional chemicals and reagents were provided by Sigma-Aldrich or other reputable suppliers.

### 2.2. Animals and Treatments

A total of twenty-four male Wistar rats weighing between 160 and 220 g were included in this study. The animals were supplied by the Pharmacy College at King Saud University. The rats had unrestricted access to food and water, and they were maintained at a standard room temperature of 23 ± 2 °C under a 12 h light/dark cycle for one week before the experiment commenced. The experimental protocol received approval from the ethics committee of King Saud University (Approval number: KSU-SE-25–18). The rats were randomly separated into four groups (*n* = 6) as detailed below:

**Group 1 (Control):** Rats received normal saline orally once daily for 14 consecutive days.

**Group 2 (N-Cur):** Rats received liposomal nano-curcumin (80 mg/kg) orally once daily for 14 consecutive days [17].

**Group 3 (CDDP):** Rats received normal saline orally once daily for 14 consecutive days and a single intraperitoneal (i.p.) injection of CDDP (7 mg/kg) on day 7 [18].

**Group 4 (CDDP + N-Cur):** Rats received N-Cur (80 mg/kg) orally once daily for 14 consecutive days and a single i.p. injection of CDDP (7 mg/kg) on day 7.

After the treatments, rats that had been fasted for 24 h were euthanized through cervical dislocation. Blood samples were collected and processed to isolate serum. Liver tissues were removed, rinsed, and weighed. Portions of the liver were homogenized in cold Tris–HCl buffer, centrifuged, and the supernatant was stored at −80 °C for the assessment of malondialdehyde (MDA), reduced glutathione (GSH), superoxide dismutase (SOD), and inflammatory markers such as tumor necrosis factor (TNF-α), interleukin-10 (IL-10), and C-reactive protein (CRP). Additional samples were placed in 10% neutral buffered formalin (NBF) for histological and immunohistochemical analysis.

### 2.3. Biochemical Assays

To evaluate liver function, serum LDH, AST, and ALT levels were measured, alongside an assessment of hepatic oxidative stress and antioxidant activities. In addition, inflammatory responses were quantified by determining hepatic levels of IL-1β, CRP, and TNF-α, while Nrf2 expression in the liver tissue was measured.

### 2.4. Histopathology and Immunohistochemistry

Liver samples were fixed in 10% neutral buffered formalin, followed by dehydration and embedding in paraffin wax. The resulting blocks were sectioned into 5 μm slices for further processing. These sections underwent deparaffinization, rehydration, and staining with hematoxylin and eosin (H&E) to facilitate histopathological examination under a light microscope. To evaluate the expression levels of β-catenin, GSK-3, and Nrf2, we employed the avidin–biotin–complex (ABC) technique as previously detailed. In brief, the sections were deparaffinized and rehydrated, then washed in tap water before being incubated in a solution of 3% hydrogen peroxide (H_2_O_2_) for 10 min. Subsequently, the sections were blocked with 5% bovine serum albumin in Tris-buffered saline (TBS) and incubated with primary antibodies for β-catenin, GSK-3, or Nrf2 at a dilution of 1:100. Following three washes in TBS, the sections were treated with appropriate secondary antibodies. After another round of washing in TBS, peroxidase activity was visualized using diaminobenzidine, with subsequent counter-staining using hematoxylin. The sections were then thoroughly washed in water, dehydrated, cleared, and mounted for examination. Quantitative analysis of protein expression via immunostaining was conducted using ImageJ (version 1.32j, NIH, Bethesda, MD, USA), with results expressed as a percentage of the control values.

### 2.5. Data Analysis

Statistical analysis was conducted utilizing one-way ANOVA and subsequently Tukey’s post hoc test in GraphPad 9 (GraphPad Software Inc., La Jolla, CA, USA). The data are represented as mean ± standard error of the mean (SEM), with statistical significance set at a *p* value of ≤0.05.

## 3. Results

### 3.1. N-Cur Attenuates CDDP-Induced Hepatic Injury

**Figure 1:**
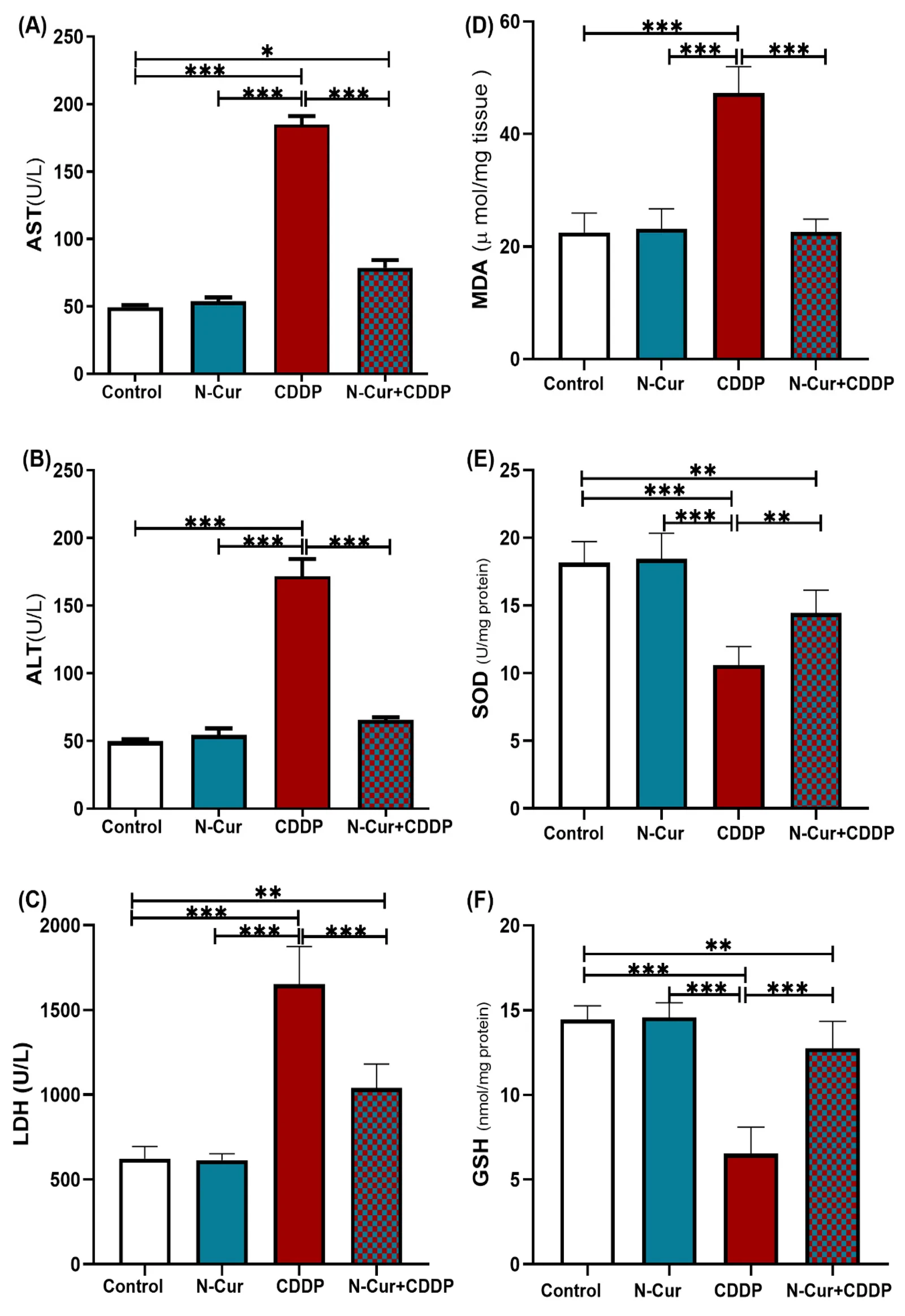
Protective effects of N-Cur on serum liver function and hepatic oxidative stress markers in CDDP-induced hepatotoxicity. (**A**–**C**) Serum liver function markers and (**D**–**F**) ameliorated hepatic oxidative stress markers. Data are presented as mean ± SEM (*n* = 6). Statistical significance is indicated as follows: * *p* < 0.05, ** *p* < 0.01; *** *p* < 0.001.

To assess the protective role of N-Cur against CDDP-induced liver damage, serum levels of liver function indicators were evaluated (Figure 1). The administration of CDDP significantly raised the levels of AST, ALT, and LDH in comparison to the control group (*p* < 0.001, Figure 1A–C), suggesting liver injury. Treatment with N-Cur significantly reduced these increases, showing notably lower levels of AST, ALT, and LDH compared to the CDDP group (*p* < 0.001). CDDP also significantly elevated hepatic lipid peroxidation (MDA; Figure 1D; *p* < 0.001) and led to a depletion of antioxidants, SOD (Figure 1E; *p* < 0.001), and GSH (Figure 1F; *p* < 0.01). N-Cur treatment countered these effects, lowering MDA levels while restoring SOD and GSH (*p* < 0.001).

**Figure 2:**
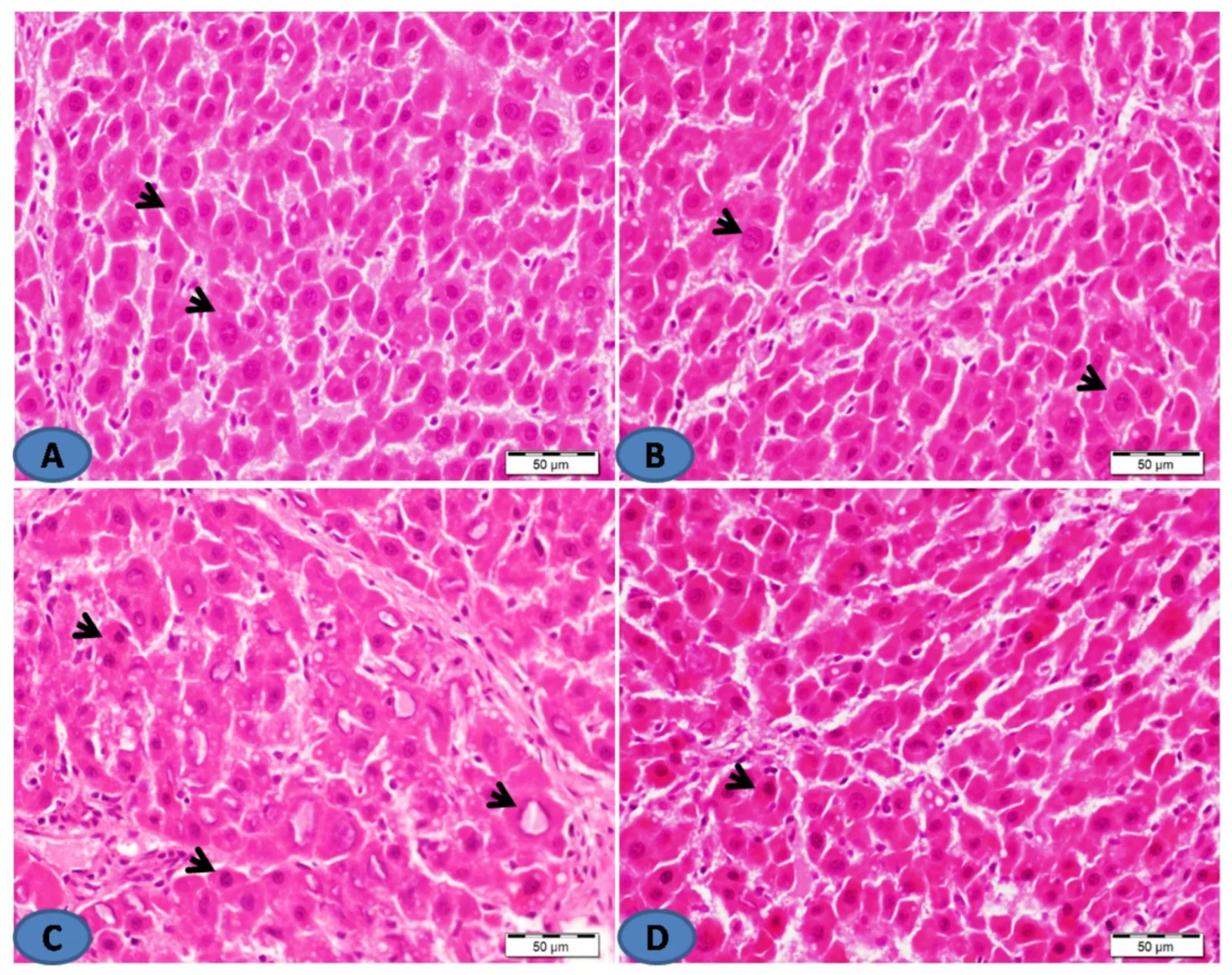
H&E-stained rat liver sections, scale bar: 100 µm. (**A**,**B**) Normal control and N-Cur-administered rats show normal hepatic architecture and cells (arrowheads) and very thin fibers restricted to portal areas (arrowheads). (**C**) The liver of a rat that received CDDP shows marked hepatic cellular degeneration (arrowheads). (**D**) The liver of a rat that received CDDP and N-Cur shows a decrease in cellular degeneration (arrowhead).

Histopathological analysis with H&E staining indicated that the hepatic structure remained intact in both the control and N-Cur groups (Figure 2A,B, respectively), featuring very thin fibers localized to portal areas. In contrast, liver tissue from rats treated with CDDP exhibited histological changes, cellular degeneration in the liver, increased thickness of fibrous tissue, and a significant presence of infiltrating inflammatory cells (Figure 2C). Importantly, N-Cur substantially mitigated these pathological issues, as evidenced by a decrease in inflammatory cell infiltration and enhancement of hepatic structure (Figure 2D).

### 3.2. N-Cur Mitigates CDDP-Induced Inflammation in Hepatic Tissue

**Figure 3:**
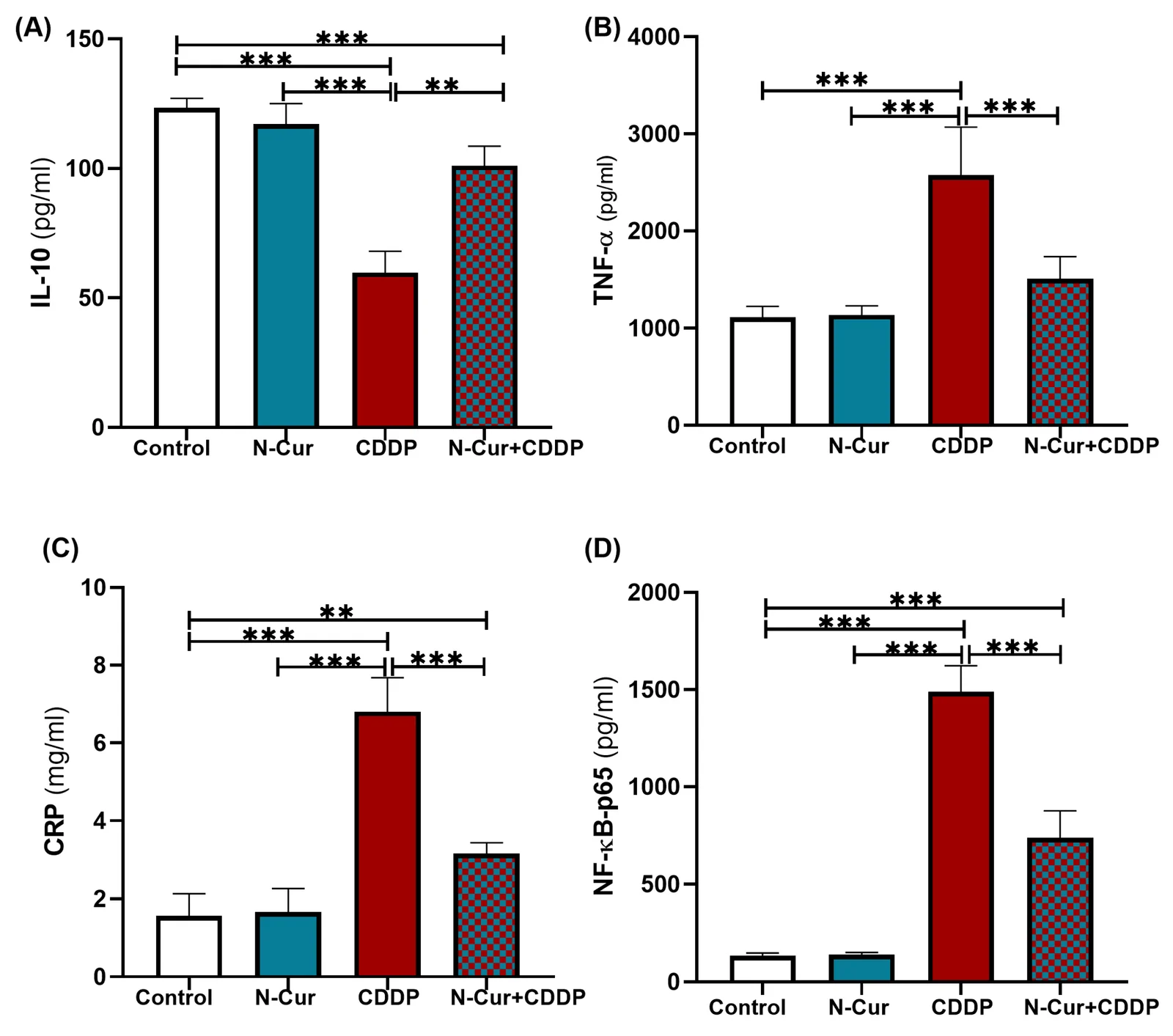
N-Cur mitigated inflammation in CDDP-treated rats. A-D N-Cur significantly decreased IL-10 (**A**), TNF-α (**B**), CRP (**C**), and NF-κB p65 (**D**) in the CDDP-administered group. Data are expressed as mean ± SEM (*n* = 6). The level of statistical significance is indicated as follows: ** *p* < 0.01; *** *p* < 0.001.

To explore the impact of N-Cur on hepatic inflammation caused by CDDP, inflammatory marker expression was evaluated using immunohistochemistry and ELISA (Figure 3). CDDP significantly decreased the levels of the anti-inflammatory hepatic biomarker IL-10 (*p* < 0.001; Figure 3A) in comparison to the control groups. CDDP significantly heightened the expression of TNF-α, CRP, and NF-κB p65 within liver tissues (*p* < 0.001; Figure 3B, 3C and 3D, respectively). N-Cur notably suppressed the expression of NF-κB p65 (*p* < 0.001), reduced the levels of TNF-α and CRP (*p* < 0.001), and increased the IL-10 level (*p* < 0.001), which supports the anti-inflammatory effects of N-Cur in cases of hepatic injury induced by CDDP.

### 3.3. N-Cur Mitigates β-Catenin/GSK-3/Nrf2 Pathway in CDDP-Induced Hepatic Injury

**Figure 4:**
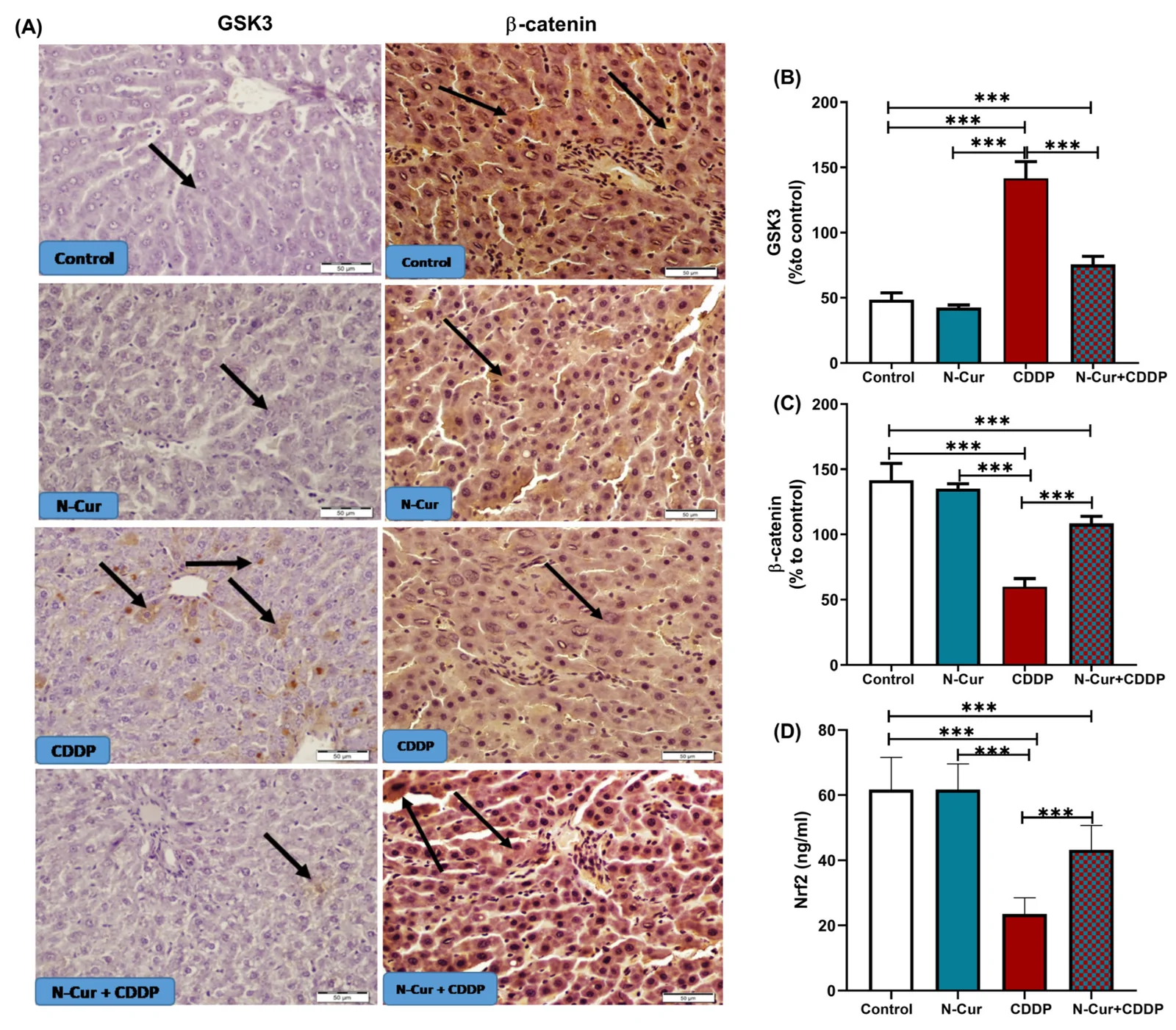
Protective effects of N-CUR on hepatic Wnt/β-catenin/GSK-3β and Nrf2 signaling pathways in CDDP-induced hepatotoxicity. (**A**) Immunohistochemical (IHC) analysis of rat liver tissue reveals distinct expression patterns for GSK-3 and β-catenin across the experimental groups. In the control and N-Cur-only groups, hepatocytes displayed normal characteristics, appearing unstained for GSK-3 (arrows) while maintaining positive cytoplasmic and nuclear reactivity for β-catenin (arrows). Conversely, CDDP administration induced significant cellular stress, evidenced by a strong GSK-3 immune reaction within the cytoplasm (arrows) and a corresponding loss of β-catenin expression in both the nuclei and cytoplasm (arrows). Notably, the co-administration of N-Cur with CDDP effectively mitigated these changes, resulting in a marked depletion of GSK-3 reactivity (arrows) and a restorative increase in β-catenin immune reactivity (arrows), suggesting a protective role for N-Cur against CDDP-induced hepatic signaling alterations. N-Cur significantly reduced GSK-3 (**B**), heightened β-catenin (**C**), and decreased hepatic Nrf2 (**D**) levels in the CDDP-administered group. Data are expressed as mean ± SEM (*n* = 6). Statistical significance is indicated as *** *p* < 0.001.

Immunohistochemistry results demonstrated that the administration of cisplatin (CDDP) resulted in a significant alteration in hepatic protein expression. Notably, there was a remarkable downregulation of β-catenin protein expression, as seen in Figure 4. In addition, Nrf2 expression was significantly reduced after CDDP treatment. In contrast, the protein expression of GSK-3 was profoundly increased compared with the control group. N-cur treatment resulted in the reversal of these consequences. Specifically, N-cur induced a marked increase in β-catenin and Nrf2 expression, while decreasing GSK-3 expression. Overall, the data showed that N-cur had a substantial impact on Wnt/β-Catenin/GSK-3 and Nrf2 signaling pathways.

## 4. Discussion

The findings of the current study demonstrate that N-Cur exerts a remarkable hepatoprotective effect against CDDP-induced liver injury as proven biochemically, histologically, and at the level of inflammatory mediators. The establishment of the CDDP-induced hepatotoxicity model was successfully demonstrated by a coordinated increase in oxidative stress and inflammatory responses, a depletion of antioxidant defenses, changes in hepatic injury markers, and characteristic histopathological changes observed in CDDP-treated animals. These findings collectively confirm the development of significant hepatic injury.

Our findings agree with earlier reports that documented the anti-inflammatory and antioxidant effects of nanoformulations of curcumin on chemotherapy-induced organ toxicities. Single-dose administration of CDDP in the current study resulted in hepatocellular damage that was largely attributed to oxidative stress, as indicated by significant elevations in liver function markers, including ALT, AST, and LDH, along with depletion of antioxidants (SOD and GSH) and elevation of MDA. These results are in line with previous work indicating that CDDP induces the leakage of hepatocellular enzymes due to reactive oxygen species generation, lipid peroxidation, and the depletion of antioxidants [19]. In line with prior studies where nano-curcumin attenuated cisplatin-induced hepatotoxicity, N-Cur administration in the current model remarkably normalized liver enzyme levels, reduced MDA, and restored antioxidant balance [20]. These findings indicate that N-Cur exerts powerful antioxidant and free radical scavenging activities, contributing to its hepatoprotective action. This supports the idea that nanoformulation of curcumin enhances its antioxidant potency, leading to greater biochemical improvements compared to conventional formulations.

The histopathological findings also support the notion that N-Cur can structurally protect liver tissue from damage induced by CDDP. In accordance with these observations, cisplatin administration in the current study resulted in hepatocellular degeneration and tissue fibrosis, whereas co-treatment with N-Cur substantially attenuated these histopathological alterations, preserving liver architecture. Comparable findings have been described in earlier studies, where nanoformulation of curcumin has been consistently associated with a marked preservation of hepatic tissue architecture and attenuation of histopathological lesions [21].

Another key feature of the present work is the central involvement of the inflammatory process in CDDP-induced hepatotoxicity. Current data demonstrate that cisplatin administration markedly upregulated the hepatic expression of inflammatory mediators, including tumor necrosis factor-alpha (TNF-α) and NF-κB p65, in addition to C-reactive protein (CRP), while reducing the anti-inflammatory cytokine interleukin-10 (IL-10). Remarkably, N-Cur counteracted these inflammatory perturbations, as evidenced by lowering the levels of TNF-α, NF-κB, and CRP while augmenting IL-10 expression. This profile is indicative of potent anti-inflammatory actions of N-Cur, which is also in harmony with previous reports in which curcumin-based nanoformulations similarly attenuated CDDP-induced inflammatory response [21]. Additionally, the hepatoprotective effect of N-Cur may involve the modulation of hepatic macrophage polarization. Curcumin has been found to promote an anti-inflammatory macrophage phenotype, which is characterized by increased levels of IL-10 and M2-associated markers such as Arg1. This could contribute to the resolution of hepatic inflammation [22]. However, since macrophage polarization and Arg1 expression were not directly assessed in the current study, this mechanism remains a plausible hypothesis that requires further investigation.

To further explore the effect of N-Cur as a protective agent against hepatotoxicity induced by CDDP, we studied the involvement of β-catenin, as previous findings showed that cisplatin administration is associated with a reduction in β-catenin expression in both the kidney and liver of rabbits. However, treatment with L-carnitine, a metabolic antioxidant, restored it to its normal level and alleviated renal and hepatic injury. These results prove the involvement of β-catenin in CDDP-mediated nephrotoxicity and present it as an appealing target of treatment [23]. One study by Lehwald, N et al. showed that β-catenin knockdown mice are more vulnerable to ischemia or reperfusion liver injury, whereas overexpressing Wnt1, an upstream signaling regulator of β-catenin, made hepatocytes resistant to the damage. In the livers of β-catenin knockdown mice, hypoxia-inducible factor (HIF)-1α signaling was diminished, an effect that was reversed in Wnt1-overexpressing hepatocytes during hypoxia and ischemia/reperfusion (I/R) [24]. In fact, β-catenin is an essential mediator that regulates hepatocyte proliferation, tissue repair, and maintenance of liver homeostasis. In addition, β-catenin activity is negatively regulated by GSK-3, which is a key regulatory kinase that phosphorylates β-catenin and marks it for ubiquitination.

Our data showed a reduction in β-catenin expression and upregulation of GSK-3 after CDDP administration, and this suggests disruption of β-catenin signaling, which contributes to enhanced inflammatory responses, impaired cellular regeneration, and increased susceptibility to hepatic injury. Conversely, treatment with N-Cur significantly restored the expression levels of β-catenin and reduced GSK-3. These findings suggest that N-Cur exerts hepatoprotective effects and impacts wnt/β-catenin/GSK-3 signaling pathways, thereby attenuating CDDP-induced inflammatory damage and promoting hepatic tissue recovery.

Collectively, the proposed mechanistic scenario of the CDDP hepatocellular insult may involve inducing oxidative stress by increasing the generation of ROS, increasing mitochondrial stress, and disrupting Wnt/β-catenin/GSK-3 and Nrf2 pathways in hepatocytes. Collectively, these changes lead to the release of damage-associated signals from compromised hepatocytes that might in turn activate Kupffer cells and recruit macrophages. Macrophage activation may subsequently amplify hepatocellular injury through TNF-α-, IL-1β-, and NF-κB-dependent inflammatory signaling. In contrast, N-Cur may attenuate this inflammatory oxidative loop through enhancement of Nrf2-associated antioxidant responses and modulation of the Wnt/β-catenin/GSK-3β axis, thereby reducing oxidative and inflammatory injury. N-Cur may also favor an anti-inflammatory macrophage phenotype, potentially associated with increased IL-10 and Arjun expression, which could further limit inflammatory signaling and support hepatic tissue repair.

## 5. Conclusions

Overall, the combined biochemical, histopathological, and immunohistochemical findings provide significant evidence supporting the hepatoprotective effects of N-Cur against CDDP-induced liver toxicity. These favorable effects of N-Cur are largely attributed to its anti-inflammatory and antioxidant effects. Moreover, the results of this study refine and extend those of previous investigations and provide integrated evidence for the beneficial effects of N-Cur at functional and structural levels and also show the effects of N-Cur on Wnt/β-Catenin/GSK-3 and Nrf2 signaling pathways.

### Study Limitations

While this study provides valuable insights into the protective effects against cisplatin-induced hepatotoxicity, several limitations should be noted. First, the absence of a positive control group utilizing conventional curcumin limits a direct comparative assessment regarding the relative therapeutic efficacy of N-Cur. Second, while key inflammatory and oxidative parameters like TNF-α and Nrf2 were quantified via ELISA, the observed alterations in the Wnt/β-catenin/GSK-3β and Nrf2 signaling pathways should be interpreted as associative findings rather than definitive evidence of direct pathway activation or cross-talk. Third, this study did not include pharmacokinetic analyses or tumor-bearing animal models to confirm that N-Cur preserves or enhances the chemotherapeutic efficacy of CDDP in vivo. Comprehensive downstream functional studies and advanced mechanistic models are required to fully elucidate these signaling interactions. Overall, this study supports the potential translational relevance of N-cur as a candidate agent for mitigating CDDP-induced hepatotoxicity, while warranting further pharmacokinetic and tumor-bearing studies to establish its clinical application.

## Data Availability

Data generated during this study are available upon request from the corresponding authors.
